# Physiotherapist’s musculoskeletal imaging profiling questionnaire: Development, validation and pilot testing

**DOI:** 10.4102/sajp.v75i1.1338

**Published:** 2019-09-04

**Authors:** Ogochukwu K.K. Onyeso, Joseph O. Umunnah, Peter O. Ibikunle, Adesola C. Odole, Canice C. Anyachukwu, Charles I. Ezema, Maduabuchukwu J. Nwankwo

**Affiliations:** 1 Department of Medical Rehabilitation, University of Nigeria, Nsukka, Nigeria; 2 Department of Medical Rehabilitation, Nnamdi Azikiwe University, Awka, Nigeria; 3 Department of Physiotherapy, University of Ibadan, Ibadan, Nigeria

**Keywords:** Imaging, Musculoskeletal, Physiotherapy, Profile, Questionnaire

## Abstract

**Background:**

Many countries have started adopting musculoskeletal imaging as part of physiotherapy practice and their educational programmes are expected to bridge the gaps in training.

**Objectives:**

To develop an instrument that can be used to explore the level and nature of training, attitude, competence and utilisation of musculoskeletal imaging among physiotherapists.

**Method:**

An exploratory sequential mixed methods design was used. An in-depth international literature search was conducted, followed by a focus group discussion (FGD). The FGD informants were recruited through maximum variation sampling. The results of the FGD and the information from relevant literature were used to draft the physiotherapist’s musculoskeletal imaging profile questionnaire (PMIPQ). The PMIPQ was then subjected to face, content and criterion validity and pilot testing. The final version of the PMIPQ consists of six domains: (A) demographic details, (B) nature of training in musculoskeletal imaging, (C) level of training, (D) attitude towards musculoskeletal imaging, (E) utilisation and (F) competence. Data were analysed using means, standard deviation, Spearman’s correlation (*ρ*) and Cronbach’s alpha (*α*); SPSS 20 software (*p* ≤ 0.05).

**Results:**

The results showed that the PMIPQ has good psychometric properties: validity and internal consistency. The test–retest reliability (*p*-value) across the domains was: C (0.973), D (0.979), E (0.842) and F (0.716).

**Conclusion:**

Physiotherapist’s musculoskeletal imaging profile questionnaire is a relevant instrument for assessing the musculoskeletal imaging profile of physiotherapists in Nigeria and in other countries with a similar scope of training and practice.

**Clinical implications:**

Musculoskeletal system imaging is a potentially useful adjunct to physiotherapists in clinical practice.

## Introduction

Physiotherapy is a health care profession that addresses the issues of human movement, functionality and quality of life (Melnick 2015). The scope of practice and span of physiotherapy training varies from one country to another (Moffat 2012). For instance, the entry-level qualification in the USA is predominantly a Doctor of Physical Therapy (DPT) (Boissonnault et al. 2014). In Nigeria, the DPT programme has been approved by the National Universities Commission (2018) as the minimum benchmark for entry-level physiotherapy education – a curriculum that deepens the contents of diagnostic imaging training (American Physical Therapy Association [APTA] 2010; Domholdt, Emery & Harris 2004; Medical Rehabilitation Therapists Board of Nigeria [MRTBN] 2009). Musculoskeletal imaging is a subspecialty of diagnostic imaging, which involves ordering and interpreting medical images of bones, joints and associated soft tissues to either identify or rule out pathologies (Reiser, Baur-Melnyk & Glaser 2008).

Musculoskeletal system imaging is indispensable to physiotherapists and has always been a component of their clinical decision-making (Domholdt et al. 2004). It has also been shown that well-trained physiotherapists can utilise diagnostic imaging appropriately as an autonomous provider or within a multidisciplinary team (Kersten et al. 2007; Moore et al. 2005).

The inadequacy in education and training of some referral sources in musculoskeletal disorders and incomplete evaluation of patients for serious pathologies prior to referral for physiotherapy have contributed to the need for physiotherapists to become better at assessment prior to managing patients (Boissonnault et al. 2014).

Physiotherapists’ competency in the utilisation of musculoskeletal imaging for clinical examination purposes was established among the US military physiotherapists 44 years ago (James & Stutart 1975).

Although this level of competency was mostly demonstrated within the military model of practice, examples also exist in civilian settings in the USA and abroad (Moore et al. 2005).

The current practice model in Nigeria is tailored towards autonomous practice, which includes unrestricted direct patient access, the ability to refer to other providers and the ability to refer for diagnostic tests (APTA 2014; MRTBN 2009). However, the Nigerian Nuclear Regulation Authority (2006) warned that diagnostic imaging should be justified by weighing its benefits against potential radiation hazards. Clinicians are advised to always consider available alternatives such as ultrasound and magnetic resonance imaging (MRI), which do not involve medical exposure. Inappropriate utilisation of imaging can lead to over-exposure of patients to radiation hazards and economic wastages (Gazelle et al. 2007; Lehnert & Bree 2010).

Various countries have legislation and regulations over diagnostic imaging (Kam 2005). In the USA, Canada, and some other countries, the *Physiotherapy Practice Act* has defined the scope of physiotherapy in diagnostic imaging (Boyles et al. 2011; Chong et al. 2015). Physiotherapists in Australia, New Zealand and the United Kingdom have diagnostic imaging referral rights within their roles as primary care providers (Prabhu & Ahmed 2017). Specifically, some states in the USA have an affirmative statement with diagnostic imaging referral right, being part of practice. In other states (such as Oregon and Arizona), radiological investigation is not included in practice acts; in New Jersey and Illinois, it is prohibited, while Colorado proposed disciplinary action against physiotherapists who request X-rays (Boyles et al. 2011). In Nigeria, the *Medical Rehabilitation Therapists Act*, M9 LFN 2004, does not prohibit physiotherapists from the utilisation of diagnostic imaging in clinical practice.

However, the use of diagnostic imaging by physiotherapists has a sound foundation for expansion in future practice and commensurate emphasis in physiotherapy education (Boyles et al. 2011; James & Stuart 1975).

Accordingly, there is a need to explore the level and nature of training, attitude, competence and utilisation of musculoskeletal imaging among physiotherapists in Nigeria. Following a search of the international literature using keywords to search databases, the authors could not identify any appropriate survey instrument, which could be used for a study such as this. Therefore, a new survey instrument, which can be used to collect comprehensive data on the musculoskeletal imaging profile of physiotherapists in Nigeria, was developed.

## Methods

An exploratory sequential mixed methods design was undertaken among registered physiotherapists in Nigeria.

A qualitative approach was used to develop the contents of the survey instrument, while a quantitative design was used to validate and pilot test the draft instrument (Dizon, Grimmer-Somers & Kumar 2011).

### Procedure

#### In-depth literature review

The literature was reviewed by searching the following databases: PEDro, MEDLINE, Embase and Google Scholar, with the keywords (physical therapy, physiotherapy, musculoskeletal imaging, diagnostic imaging, profile and questionnaire) that made up the title of our study. We did not find any comprehensive instrument that could be used to explore the nature of training, attitude, competence and utilisation of musculoskeletal imaging among physiotherapists. However, the following bodies of literature were found relevant in drafting the initial version of the instrument: physiotherapists’ perceptions and use of medical imaging information in practice (Little & Lazaro 2006); Ontario physiotherapists’ opinions on an expanded scope of practice and ordering diagnostic imaging (Chong et al. 2015); and studies by Sak-Ocbina et al. (2016), Boissonnault et al. (2014) and Potter, Cairns and Stokes (2012). A step-by-step approach for the development, validation, pilot testing and implementation of an online profiling questionnaire was adopted from the works of Dizon et al. (2011), Streiner and Norman (2008), Boynton (2004), Boynton and Greenhalgh (2004) and Andrews, Nonnecke and Preece (2003).

#### Focus group discussion

Maximum variation sampling was used to set up a focus group discussion (FGD). Ten key informants (three women and seven men) were recruited across all the possible demographic variables of the population of the study. These key informants were identified and contacted through the network of the Association of Clinical and Academic Physiotherapists of Nigeria (ACAPN). The inclusion criterion was as follows: participant must be a registered physiotherapist, who is currently licensed and practising in Nigeria for at least 2 years. Potential participants were informed of the study objectives and the mode of the meetings. They granted their individual informed consent and they were added to a social media (WhatsApp) forum created for that purpose. This technological innovation of social media meeting reduces the challenges of a traditional focus discussion such as geographical barriers, mandatory physical presence, convenient timing, meeting logistics and cost, the burden of transcribing the audio recording and loss of man-hours. One of the key informants facilitated the sessions, while one of the authors tracked and highlighted all the points raised. Another author and a software developer who later designed the online version of the instrument were also added to the forum as observers.

Open-ended questions were asked to explore the informants’ perspectives of survey content. The core questions were: (1) what are the important demographic characteristics of physiotherapists in Nigeria, relevant to the present study?, (2) what is the nature, content and amount of musculoskeletal imaging training received by physiotherapist in Nigeria during undergraduate, internship, postgraduate and workshops?, (3) what factors drive the attitude of physiotherapists in Nigeria towards musculoskeletal imaging?, (4) what factors influence Nigerian physiotherapists’ utilisation of musculoskeletal imaging? and (5) what are the imaging modalities a physiotherapist needs to be competent in to optimise his or her clinical practice?

Afterwards, all the comments from the FGD were collated and reposted in the forum until all the key informants confirmed that the issues raised during the discussions had been captured. The outcome of the FGD was given to a panel of two independent reviewers (university senior lecturers) to harmonise (Dizon et al. 2011; Patton 2002) and two of the authors liaised with the panel and retrieved the results.

#### Development of the draft survey instrument

Information from the reviewed literature and FGD were used to draft the physiotherapists’ musculoskeletal imaging profiling questionnaire (PMIPQ). The initial draft of the instrument was sent to the members of the focus group to check for congruence with their recall of the FGDs. The comments from the participants were integrated into the draft before sending it for expert validation; this approach ensured the completion of the triangulation processes. Dizon et al. (2011) recommended a dual triangulation method for this type of study to ensure the validity of findings from all sources of information. Our study utilised both investigator and data triangulation methods.

### Face, content and criterion validation

The draft questionnaire was subjected to face and content validity. A six-man validation panel of experts was selected based on the Hoffmann expertise proficiency scale (Chi 2006). The panel consisted of six physiotherapists (three professors, a reader, a senior lecturer and a clinician [a retired assistant director of physiotherapy with over 35 years of experience]) who did not participate in the FGD.

Their areas of expertise were physiotherapy education, musculoskeletal physiotherapy and questionnaire development. The experts had at least 15 years post-qualification experience in (academic or clinical) practice and with publications in related fields of physiotherapy. The panellists were sent an anonymous blind carbon copy email, seeking their individual informed consent to participate in the validation process.

Following their permission, they received the objectives of the study and a copy of the draft questionnaire, through an email. Then, a Delphi method of information exchanges (facilitated through the anonymous email address: pmipqresearcher18@yahoo.com) was employed until the validation panellists reached consensus that the questionnaire was appropriate for the study (Dizon et al. 2011). The Delphi method was utilised because it is a means of collecting and combining the comments or opinion of a group of experts who do not meet face to face, using information exchanges (Campbell et al. 2002).

The panellists were required to check the relevance of the questions in line with the questionnaire domains (content validity) and to comment on the orderliness of the questions and response options (face validity). The experts were asked to comment on and rate the questionnaire’s length, whether it was easy to comprehend (language and terms), adequacy of content, chronology and clarity of instructions, questions and answer options, using a three-point scale (1 – not appropriate, 2 – neutral and 3 – appropriate).

The responses from the panellists were collated and the appropriate correction was made. The revised instrument was returned to the panel of experts for further review, feedback and consensus. Exchanges were done twice before consensus was reached. The validated instrument covered six main domains labelled as parts A–F (Table 1).

**TABLE 1:** Contents of the draft survey instrument.

Themes	Description
Part A. Demographic details and general Information	Intended to obtain demographic details, licence renewal history, years of practice, region of practice, practice setting, employment cadre, specialty of interest, continuous professional development and educational background.
Part B. Nature of training in musculoskeletal imaging	Intended to obtain entry points, methods, duration, personnel employed and hands-on experiences for undergraduate, internship, workshop and postgraduate trainings.
Part C. Level of training of respondents in utilisation of musculoskeletal imaging	Intended to obtain the level of training of respondents on interpretation and utilisation of results from the following musculoskeletal imaging modalities (X-ray, MRI, CT scan, ultrasound, bone scan and DEXA).
Part D. Attitude of respondents to musculoskeletal imaging	Intended to obtain the attitude of respondents towards the use of musculoskeletal imaging in clinical practice.
Part E. Level of utilisation of musculoskeletal imaging results in clinical practice	Intended to obtain the level of utilisation of musculoskeletal imaging results in clinical practice (utilisation of X-ray, MRI, CT scan and bone scan results, ordering DEXA before manipulation in geriatrics, performing musculoskeletal ultrasonography), referral rights and others.
Part F. Level of competence in interpretation of musculoskeletal imaging results	Intended to obtain the level of competence of physiotherapists in interpretation of musculoskeletal imaging results, specifically X-ray, MRI, CT scan, ultrasound, bone scan and DEXA.

MRI, magnetic resonance imaging; CT, computed tomography; DEXA, dual-energy X-ray absorptiometry.

During pilot testing, the instrument was further tested for criterion validity. Criterion validity is defined as the correlation of a scale with an accepted instrument or measure (Dizon et al. 2011). Although the psychometric properties of the questionnaire by Chong et al. (2015) have not been reported, the authors adopted section B (utilisation), D1 (attitude) and E2 (competence) of the instrument as the criterion for this study because of the similarity in construct. The authors employed a convergent criterion validity approach. The criterion was concurrently administered with the new questionnaire during the pilot testing, and the scores of both instruments were correlated. Nonetheless, construct validity of the instrument was not tested because the instrument was not designed to measure a specific hypothetical construct (Dizon et al. 2011). The PMIPQ is a simple survey, designed to collect data from participants on their level of training in diagnostic imaging, their attitude, competence and utilisation of the modalities in clinical practice.

### Pilot testing

A mixed method of conducting pilot testing by using both paper and online questionnaires was adapted from Dizon et al. (2011). Thirty-one physiotherapists practising in Southern Nigeria, who did not participate in the FGD or validation processes, were recruited through convenient sampling. The instrument was printed and self-administered alongside a criterion questionnaire, retrievable from https://doi.org/10.3138/ptc.2014-09 (Chong et al. 2015). After 1 week of completion of the paper questionnaire, the online version of the instrument (without the criterion) was sent to the same respondents by emailing them the instrument’s uniform resource locator (URL): http://survey.spanatech.com. Text messages were sent out twice (at 3 days intervals) to remind the respondents to complete the questionnaire within 1 week.

Moreover, the online survey tracked and recorded the time it took each respondent to complete the questionnaire. The paper version contained a checklist (Table 2) which allowed the respondents to critique the instrument and to comment on areas where they sought clarification or changes. The data collected during piloting were used to analyse the criterion validity, test–retest reliability and internal consistency of each domain of the instrument where applicable.

**TABLE 2:** Checklist for physiotherapist’s musculoskeletal imaging profiling questionnaire: Summary of the respondents’ opinion on the characteristics of the instrument.

S/N	Questionnaire characteristic	Not appropriate	Neutral	Appropriate	Remarks
1	Relevancy	-	-	X	-
2	Length	-	X	-	-
3	Simplicity or easy to comprehend language and terms	-	-	X	-
4	All-inclusive	-	-	X	-
5	Adequacy of content	-	X	-	-
6	Chronology or systematic arrangement	-	-	X	-
7	Self-explanatory	-	-	X	-
8	Clarity of instructions, questions and answer options	-	-	X	-
9	Easy to fill	-	-	X	-
10	(Other comments on areas you seek clarification or changes)	-	-	-	-

**Instruction:** Kindly complete this checklist based on your assessment of this instrument. Use ‘X’ to mark the option that depicts your opinion about the questionnaire.

### Statistical analysis

The responses to the paper version of the instrument were manually collated in a Microsoft Excel spreadsheet, while the online version was self-collated in a database and was downloaded in the same file format. Computer-based analysis of the data was performed using SPSS 20 software (SPSS, Chicago, IL, USA). The time spent in completion of the online version of the instrument was analysed with a mean ± standard deviation. Criterion validity (convergence) and reliability of the instrument (test–retest; paper scores vs. online scores) were analysed with Spearman’s correlation coefficient (*ρ*). The internal consistency of the instrument was tested with Cronbach’s alpha (*α*). Where applicable, the decision rule was set at *p* ≤ 0.05, *α* ≥ 0.5.

### Ethical consideration

Ethical approval was obtained from the Health Research and Ethics Committee of the Faculty of Health Sciences and Technology, Nnamdi Azikiwe University, Nnewi campus, Nigeria, prior to the commencement of the study (reference number: ERC/FHST/NAU/2018/193). The objectives of the study were clearly explained on the informed consent form attached to the questionnaire and endorsed by each participant.

## Results

The first draft of the instrument consisted of the major themes that were raised from the FGD (Table 1). It was organised into six domains and sent to the selected team of experts for validation. The outcome of the validation processes led to the modification in chronology, the addition of new questions and expansion of some response options. For instance, the panellists suggested that ‘not applicable’ should be included among the answer options for the items in part B. This was because some of the questions were found to be not applicable to all the potential respondents.

The face and content validated instrument was divided into (parts A–F) six domains as follows: (A) 17 questions on demographic details which include age, gender, marital status, licence renewal history, years of practice, the region of practice, practice setting, employment cadre, the specialty of interest, continuous professional development and educational background; (B) 25 questions on nature of training in musculoskeletal imaging which includes entry points, methods, duration, personnel employed and hands-on experiences for undergraduates, internship, workshop and postgraduate training; (C) seven questions on the level of training in the interpretation of musculoskeletal imaging results; (D) eight questions on the attitude of physiotherapists towards musculoskeletal imaging; (E) seven questions on the utilisation of musculoskeletal imaging results in clinical practice and (F) six questions on the level of competence of physiotherapists in the interpretation of musculoskeletal imaging results.

In total, the questionnaire was made up of 70 items. Parts A and B are multiple choices or dichotomous questions (yes or no). This aspect was designed for descriptive purposes and did not have a specific scoring system. The remaining domains (parts C–F) were designed as a five-point Likert scale (1 – lowest to 5 – highest score). Specifically, the range of score for the domains was: part C (6–30), part D (8–40), part E (7–35) and part F (6–30).

Another essential component of the methodology was piloting the PMIPQ on representatives of the study population (Dizon et al. 2011). Thirty-one physiotherapists (12 women and 19 men) averagely aged 33.81 ± 5.04 years and practice experience of 7.58 ± 5.16 years participated in the pilot survey. The average time taken to complete the online version of PMIPQ was 9.56 ± 1.51 min. The amount of time taken for completing the hard copy version of the instrument could not be analysed because it was co-administered with a criterion and a short checklist (Table 2) – to critique the general properties of the questionnaire.

Information from the checklists showed that a few respondents requested a revision of the language structure of part B for clarity. Nonetheless, the summary of the checklist (Table 2) showed that the PMIPQ was relevant, simple, all-inclusive, chronological, self-explanatory and easy to complete. The instrument has good psychometric properties: face, content and criterion validity (Table 3). The internal consistency (*a*-score) and the test–retest reliability (*ρ*-score) across the domains were: part C (*α* = 0.731, *ρ* = 0.973), part D (*α* = 0.737, *ρ* = 0.979), part E (*α* = 0.446, *ρ* = 0.842) and part F (*α* = 0.796, *ρ* = 0.716).

**TABLE 3:** Psychometric properties of the physiotherapist’s musculoskeletal imaging profiling questionnaire.

Psychometrics	Part A Demographics	Part B Nature of training	Part C Level of training	Part D Attitude	Part E Utilisation	Part F Competence
Face validity	Appropriate	Appropriate	Appropriate	Appropriate	Appropriate	Appropriate
Content validity	Appropriate	Appropriate	Appropriate	Appropriate	Appropriate	Appropriate
Criterion validity (*ρ*) (convergence)	Not applicable	Not applicable	0.151	0.371	0.515*	0.481*
Internal consistency (*α*)	Not applicable	Not applicable	0.731*	0.737*	0.446	0.796*
Reliability (*ρ*)	Not applicable	Not applicable	0.973*	0.979*	0.842*	0.716*

α* = Cronbach’s alpha ≥ 0.5; p* = Spearman’s correlation coefficient significant at *p* ≤ 0.05.

## Discussion

Our study explored the current musculoskeletal imaging practice among physiotherapists in Nigeria. We could not find any instrument in the literature that could address the objectives of our proposed study. Therefore, the development of the PMIPQ was undertaken. The questionnaire was designed, validated and piloted in adherence with the general guidelines for development and implementation of (face-to-face and online) dual administration mode surveys (Adje 2016; Andrews et al. 2003; Dizon et al. 2011; Potter et al. 2012). The PMIPQ was developed to collect data on the nature of training, attitude, competence and the level of utilisation of musculoskeletal imaging among physiotherapists in Nigeria and countries with similar education and practice models. This instrument is relevant in light of the growing influence of musculoskeletal system imaging in physiotherapy practice worldwide (Boissonnault et al. 2014).

The scope of practice and span of physiotherapy training vary from one country to another (Moffat 2012). Similarly, the curricula and diagnostic imaging contents vary among training institutions within and across countries (Boissonnault et al. 2014). Physiotherapy entry-level qualification in some countries does not involve internship training (Onigbinde 2006). In Nigeria, the post-entry-level internship programme is mandatory for all physiotherapists since its commencement in 1994. Therefore, the information on the nature of musculoskeletal imaging training during the internship was not applicable to respondents who had graduated earlier. Accordingly, the result of the pilot survey led to the adjustment of PMIPQ to accommodate respondents who did not have internship training.

All the domains (parts) of the online version of the instrument were programmed as compulsory fields except for part B where a respondent can choose the ‘not applicable’ option and move on to the next subsection. The software ensures that incomplete questionnaires cannot be submitted; rather, the missing fields will be highlighted for the respondent to complete them before submission. There is no question that this innovation reduces the problem of incomplete data – a major challenge in questionnaire-based surveys (Andrews et al. 2003). The PMIPQ was reported to be self-explanatory, easy to administer and appealing to respondents (Table 2). Boynton (2004) has stated that poor understanding of a questionnaire can lead to low response rates or incomplete data.

The results of our study showed a positive correlation between the criterion and all the tested domains of the PMIPQ. However, part E (*ρ* = 0.515, *p* = 0.006) and F (*ρ* = 0.481, *p* = 0.039) have a statistically significant and stronger positive correlation with the criterion. This outcome was expected because the criterion has a closer construct with parts E and F. The criterion was specifically designed to obtain opinions on competence and level of utilisation (of imaging modalities among physiotherapists) which corresponds with the objectives of parts E and F of the PMIPQ, respectively.

Nonetheless, all the domains of the online version of the PMIPQ showed an acceptable level of internal consistency except part E, which obtained responses on the level of utilisation of imaging studies. The value *α* = 0.446 fell below the cut-off value of *α* = 0.5. This can be explained by the fact that there is no standard practice act regarding the utilisation of imaging among physiotherapists in Nigeria. Instead, hospitals have diverse internal policies on the scope of practice. However, there was a high reliability score across all the domains (Table 3). Reliability was obtained by a pair-wise correlation between the respondents’ scores on the paper and the online versions of the PMIPQ.

The emphasis PMIPQ laid on ultrasonography as content in physiotherapy training and practice is noteworthy.

This is because ultrasound is a promising area for future practice. It has the comparative advantage of being portable, inexpensive and safe, as well as being a non-ionising-radiation-based musculoskeletal imaging modality (Boyles et al. 2011; Chong et al. 2015; Potter et al. 2012).

This study combined a qualitative and quantitative approach in analysing the outcome of the pilot testing; a similar study that reported the processes of development of a new instrument relied only on a qualitative approach (Dizon et al. 2011). Data entry and coding are recommended components of pilot testing as this allows troubleshooting of possible problems in data management and analysis (Boynton 2004). Hence, this methodology appears unique and may interest other researchers who may wish to carry out a similar study.

### Final instrument

After merging all comments from the developmental steps (Table 4), the PMIPQ was produced (see Appendix 1). The PMIPQ is currently relevant to Nigeria and could be useful in other countries with similar scopes of practice. Researchers who may want to adopt the instrument for an online survey should ensure that the instrument is presented as a single web page with a clear separation of the parts using a prominent bold-face font.

**TABLE 4:** Practical applications in designing profile questionnaires.

Steps	Purpose	Who are involved?
Initial scoping	To check the availability of an appropriate instrument which matches the objectives of an intended study If no instrument is available, identify ways and possible sources of information on how to develop an instrument which will match the intended objectives of a study	Authors Researchers
Focus group interviews	To explore key areas of concern in designing the instrument	Key informants and researchers
Validation using the Delphi technique	To ensure that the instrument measures what it intends to measure To collect highly generalisable data from the answers to be collected	Experts in relevant areas of the study
Pilot testing	To ‘trial’ the survey and identify possible problems to be encountered and allow troubleshooting to address the problems	Participants representative of the sample population

*Source*: Adapted from Dizon, J.M.R., Grimmer-Somers, K. & Kumar, S., 2011, ‘The physical therapy profile questionnaire (PTPQ): Development, validation and pilot testing’, *BMC Research Notes* 4, 362. https://doi.org/10.1186/1756-0500-4-362

## Conclusion

This study has provided a relevant instrument for assessing the musculoskeletal imaging profile of physiotherapist in Nigeria and abroad. The PMIPQ has acceptable psychometric properties, the design is comprehensive and mode of administration is innovative and appealing to respondents.

## Appendix 1

Physiotherapists Musculoskeletal Imaging Profiling Questionnaire (Nigerian Online Version)
